# Gelatinases, endonuclease and Vascular Endothelial Growth Factor during development and regression of swine luteal tissue

**DOI:** 10.1186/1471-213X-6-58

**Published:** 2006-11-30

**Authors:** Luciana Andrea Ribeiro, Maria Elena Turba, Augusta Zannoni, Maria Laura Bacci, Monica Forni

**Affiliations:** 1 Department of Veterinary Morphophysiology and Animal Production (DIMORFIPA), University of Bologna, Via Tolara di Sopra 50, 40064, Ozzano dell'Emilia, Bologna, Italy

## Abstract

**Background:**

The development and regression of corpus luteum (CL) is characterized by an intense angiogenesis and angioregression accompanied by luteal tissue and extracellular matrix (ECM) remodelling. Vascular Endothelial Growth Factor (VEGF) is the main regulator of angiogenesis, promoting endothelial cell mitosis and differentiation. After the formation of neovascular tubes, the remodelling of ECM is essential for the correct development of CL, particularly by the action of specific class of proteolytic enzymes known as matrix metalloproteinases (MMPs). During luteal regression, characterized by an apoptotic process and successively by an intense ECM and luteal degradation, the activation of Ca^++^/Mg^++^-dependent endonucleases and MMPs activity are required. The levels of expression and activity of VEGF, MMP-2 and -9, and Ca^++^/Mg^++^-dependent endonucleases throughout the oestrous cycle and at pregnancy were analyzed.

**Results:**

Different patterns of VEGF, MMPs and Ca^++^/Mg^++^-dependent endonuclease were observed in swine CL during different luteal phases and at pregnancy. Immediately after ovulation, the highest levels of VEGF mRNA/protein and MMP-9 activity were detected. On days 5–14 after ovulation, VEGF expression and MMP-2 and -9 activities are at basal levels, while Ca^++^/Mg^++^-dependent endonuclease levels increased significantly in relation to day 1. Only at luteolysis (day 17), Ca^++^/Mg^++^-dependent endonuclease and MMP-2 spontaneous activity increased significantly. At pregnancy, high levels of MMP-9 and VEGF were observed.

**Conclusion:**

Our findings, obtained from a precisely controlled in vivo model of CL development and regression, allow us to determine relationships among VEGF, MMPs and endonucleases during angiogenesis and angioregression. Thus, CL provides a very interesting model for studying factors involved in vascular remodelling.

## Background

The growth rate of corpora lutea (CL) immediately after ovulation is 4- to 20-fold more intense than that of some of the most malignant human tumours and is sustained by the formation of new blood vessels from capillaries of the theca interna compartment [1]. Actually, 85% of the dividing cells in the primate corpus luteum during the early luteal phase are endothelial cells [2]. The duration of this intense angiogenic phase in the corpus luteum varies among species, but appears to be completed by day 6 of the luteal phase in primates when capillaries surround almost all luteal cells and capillary dilation is evident [3]. Vascular Endothelial Growth Factor (VEGF) effect on endothelial cells mitosis and differentiation is considered fundamental in vascular bed development. We have previously determined the ability of swine granulosa and theca cells of growing follicles as well as luteal cells to produce VEGF [4,5].

After the stimulation of endothelial cells mitosis for neovascular tubes development, the remodelling of extracellular matrix is necessary and different proteolytic enzymes are involved [6]. The extracellular matrix (ECM) has become recognized as a key regulatory component in cellular physiology, providing an environment for cell migration, division, differentiation, anchorage, and in some cases, an ultimate fate between cell survival or cell death [7]. The highly regulated control of ECM turnover and homeostasis occurs, in part, by the action of a specific class of proteolytic enzymes known as the matrix metalloproteinases (MMPs). The MMPs and their associated endogenous inhibitors act in concert to control aspects of reproductive function. In the ovary and uterus, the MMP system has been postulated to regulate all the dynamic structural changes that occur throughout the oestrous cycle [8].

To allow repeated opportunities of fertilization, the duration of CL life is rigorously programmed, in fact, after a first period of rapid growth the tissue becomes stably organized and prepares to switch to the phenotype required for its next apoptotic regression [9,10]. Five to ten grams of luteal tissue growth and disappear at each ovarian cycle (21 days in sow).

The switch between growth and regression is mainly regulated by LH (luteinising hormone – luteotrophyn) and PGF2α (prostaglandin F2α – luteolysin) and their receptors balance as well as by cytokines, growth factors, apoptosis/oncogenes related factors and plasminogen activator/matrix metalloproteinase activators and inhibitors [10-12]. If fertilization has not occurred, or implantation was unsuccessful, or the pregnancy ends, luteolysis is initiated whereby the CL rapidly loses its progesterone-producing ability followed by degradation of luteal tissue [13]. Luteal regression is thought to occur through apoptotic [9,14] and proteolytic [15] mechanisms; however, the molecular mechanisms underlining this event are not well characterized [8,16]. Apoptosis requires a wide extracellular matrix remodelling [17] as well as the action of a Ca^++^/Mg^++^-dependent endonuclease, that is developmentally regulated in rat luteal cell nuclei [18], leading to the degradation of genomic DNA into discrete oligonucleosome fragments. Therefore angiogenesis and apoptosis and their regulation in the CL play a fundamental role in the maintenance of reproductive performances. Furthermore, the ovary is the unique organ where strictly regulated tissue hyperplasia and regression take place in a cyclic manner under physiological conditions.

This study was aimed at characterizing the temporal expression pattern of VEGF, MMP-2, MMP-9 and Ca^++^/Mg^++^-dependent endonuclease throughout the lifespan of swine CL and investigating whether the expression levels of those molecules are related to CL functional stage.

## Results

### CL functional stage assessment

**Figure 1:**
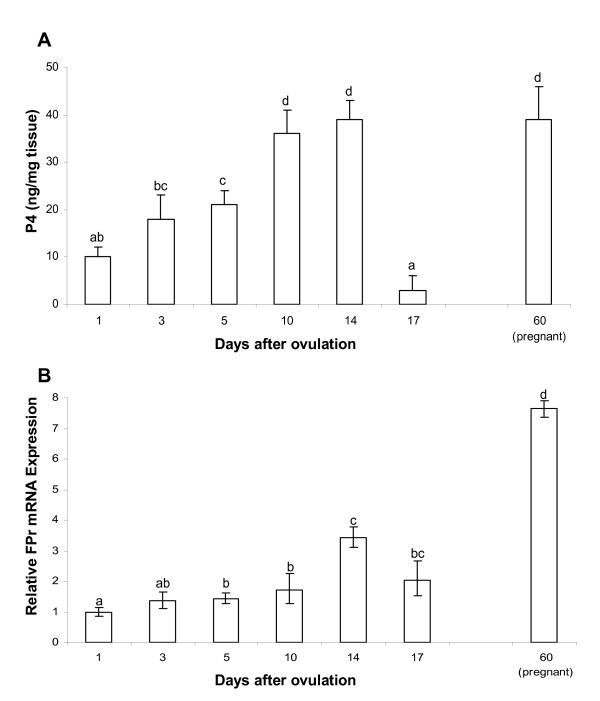
P4 and FPr mRNA levels in swine CL during different luteal phases and pregnancy. A: Changes in P4 levels (mean ± SEM). B: Changes in FPr mRNA expression in relation to day 1; error bars represent the range of relative expression. The statistical analysis were based upon the mean of 5 CLs per animal (n = 4/time point). Different letters represent significant differences (p < 0.05).

Both luteal progesterone (P4) concentration and prostaglandin F2α receptor (FPr) mRNA expression followed typical swine luteal phase patterns. P4 concentrations increased gradually during the formation of CL, reaching the highest level during the mid-late phase (days 10 and 14), and then declined significantly on day 17 (Fig 1A). Similarly, FPr mRNA levels increased gradually to a greater extent on day 14 (3.4-fold in relation to day 1, Fig 1B). At pregnancy, high levels of P4 and FPr mRNA (7.7-fold increase in relation to day 1) were observed.

### DNase activity assay

**Figure 2:**
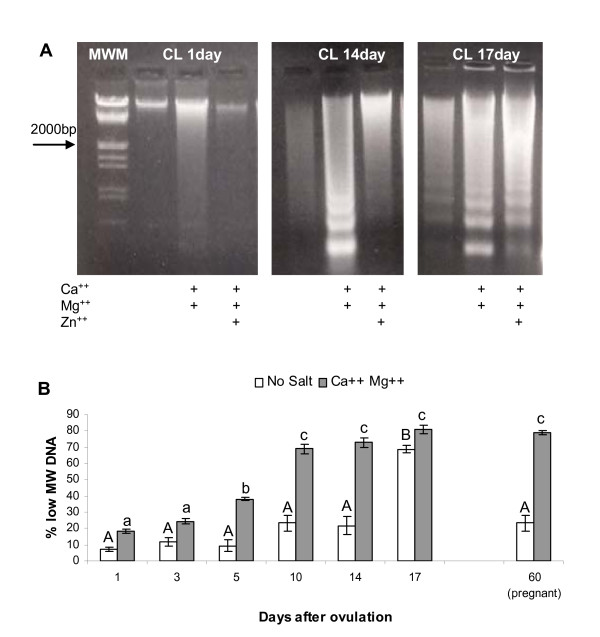
A: Representative electrophoresis profile of low molecular weight DNA from CL at day 1, 14 and 17. Each lane contains 10ug of DNA extracted from luteal nuclei after treatment with different cations. B: Nuclease activity in swine CL nuclei during different luteal phases and pregnancy. For each time point only No Salt (open bars) and Ca^++^+Mg^++ ^(closed bars) samples were presented. Data represent means ± SEM of percentage of low molecular weight DNA (≤ 2000 bp). The statistical analysis were based upon the mean of 5 CLs per animal (n = 4/time point). Different capital and small letters represent significant differences (p < 0.05) for No Salt and Ca^++^+Mg^++ ^groups, respectively.

A regulated Ca^++^/Mg^++^-dependent endonuclease expression and activity were detected in swine CL. Fig 2A illustrates the Ca^++^/Mg^++^-dependent endonuclease activity found in luteal nuclei obtained at days 1, 14 and 17. A marked activity was obtained with both Ca^++ ^and Mg^++ ^whereas the addition of Zn^++ ^inhibited almost completely the enzyme activity.

The amount of activable DNase gradually increased during the oestrous cycle, maintaining high at pregnancy. Moreover, a high level of spontaneous apoptosis in the control group (No Salt) at day 17 was also observed (Fig 2B).

### VEGF content

**Figure 3:**
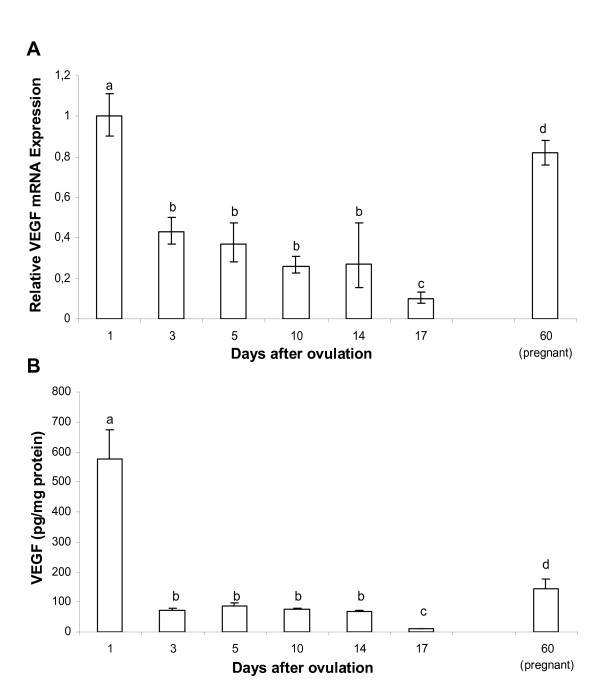
VEGF mRNA and protein levels in swine CL during different luteal phases and pregnancy. A: changes in VEGF mRNA expression in relation to day 1; error bars represent the range of relative expression. B: changes in VEGF content (mean ± SEM). The statistical analysis were based upon the mean of 5 CLs per animal (n = 4/time point). Different letters represent significant differences (p < 0.05).

Both VEGF mRNA and protein presented similar patterns of expression throughout the oestrous cycle. The highest values of VEGF were observed immediately after ovulation, decreasing significantly at day 3, and remaining unchanged during the mid-luteal phase. At day 17, a second significant drop was observed. At pregnancy VEGF mRNA and protein levels were intermediate between those of day 1 and 3 (Fig 3).

### MMPs activity assay

**Figure 4:**
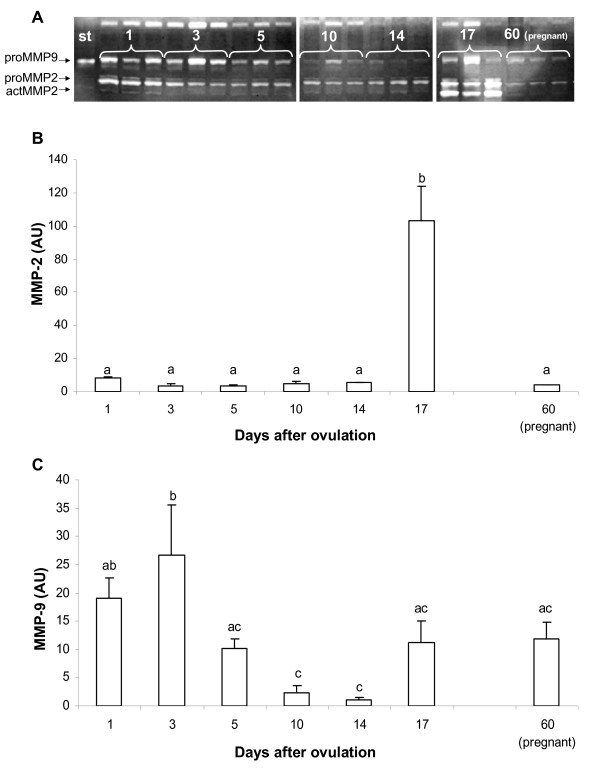
A: Representative gelatin substrate zymography gel showing gelatinase activity in swine CL during different luteal phases and pregnancy. B: Relative abundance of MMP-2 as determined by densitometric analysis expressed as means ± SEM. C: Relative abundance of MMP-9 as determined by densitometric analysis expressed as means ± SEM. The statistical analysis were based upon the mean of 5 CLs per animal (n = 4/time point). Different letters represent significant differences (p < 0.05).

Three distinct bands of gelatinase activity corresponding to latent MMP-9 (proMMP9), latent MMP-2 (proMMP2) and active MMP-2 (actMMP2) were evidenced in the swine CL (Fig 4A). Gelatinolytic activities for both latent and active forms of MMP-2 were considered together for the analysis. The activities of MMP-2 and MMP-9 changed over the luteal phase. Constant levels of MMP-2 activity were observed during CL formation, increasing significantly in late (day 17) luteal phase (Fig 4B). In contrast, MMP-9 activity peaked in the early (days 1–3) and late (day 17) luteal phase, showing the lowest values in the midluteal phase (days 10–14 – Fig 4C). At pregnancy, basal levels of MMP-2 and high levels of MMP-9 were observed.

## Discussion

The CL is a transient endocrine gland, which is formed from the remnants of an ovulated follicle [13]. During this process, a capillary network invades from the theca tissues into the granulosa layers through a dynamic angiogenesis process such that after its formation, the CL is one of the most vascularised organs in the body [16]. During VEGF driven angiogenesis, microvascular endothelial cells produce gelatinases (MMP-2 and -9), which breach the perivascular basement membrane and allow endothelial cells to migrate outward through the extracellular space [19-21]. MMPs are also involved, together with Ca^++^/Mg^++^-dependent endonuclease, in apoptotic tissue remodelling at luteolysis.

In this study, proteases with gelatinolytic activity that is consistent with MMP-9 and MMP-2 family members [7] were identified in CL collected during the oestrous cycle and at pregnancy. The gradual increase of FPr mRNA expression, peaking on day 14, together with the accumulation of Ca^++^/Mg^++^-dependent endonuclease, confirms the competence acquired by the luteal tissue around this moment to rapidly react to PGF2α and to initiate the regression. These characteristics are maintained in the pregnant CL which is always sensible to PGF2α. Moreover, elevated concentrations of P4 at day 14 corroborate the CL ability to sustain an eventual pregnancy.

Members of MMP-system may be involved in several of the proteolytic events that take place in the ovary during the reproductive cycle [16]. MMP-2 and MMP-9 have previously been detected by zymography in homogenates of rat ovaries [22,23], and in CL of bovine [24], human [15], primates [25], mouse [26], ovine [27] and swine [17]. The main role proposed for MMPs concerns the tissue remodelling associated to luteolysis.

Our findings demonstrated the presence of MMP-2 and MMP-9 in the corpus luteum during different ovarian phases. MMP-2 activity was basal during early-mid luteal phase and at pregnancy while was maximal during luteolysis. MMP-2 plays a role in various tissue remodelling processes, including trophoblast invasion [28] and tumour cell motility [20]. However, a persistence of MMP-2 activity throughout the CL formation supports suggestions that tissue remodelling continues throughout CL development and maintenance.

Concerning MMP-9 activity, high levels were observed during early luteal phase, luteolysis and pregnancy. MMP-9 is probably involved in the extensive tissue remodelling that occurs during CL formation, when extracellular material, composed primarily of follicular elements that represent a basement membrane-type ECM, is removed [15,29]. This clearance may create a more hospitable or spacious environment for the subsequent rapid proliferation and intermingling of luteal cells [21,30] and development of luteal ECM [31]. This hypothesis is supported by the findings that it is the primary metalloproteinase detected in follicles explants [32]. In addition, MMP-9 is the major MMP secreted into the culture medium of luteinised bovine [33] and human granulosa cells [34].

A different activity profiles for both MMP-2 and MMP-9 were also observed in human [15] and bovine [21] corpus luteum. In the porcine CL, mRNA expression for MMP-2 and MMP-9 was low during early luteal period (days 6–8 of the oestrous cycle), increasing significantly during midluteal period (days 9–11), and reaching the highest values during the late luteal period (days 13–15) [17]. These results agree well with ours, considering that the authors did not studied the earliest period of oestrous cycle (days 1–5 after ovulation), period in which we verified major alterations of MMP-9 activity.

In the early luteal phase, we also detected the highest levels of VEGF. As expected, VEGF mRNA and protein were detected during all days of the oestrous cycle and showed similar pattern of expression. High levels were verified immediately after ovulation, concomitantly with the intense luteal vascular growth and CL formation. Thus supporting the role of VEGF in the angiogenesis of the newly formed CL. Changes of VEGF levels in CL during the oestrous cycle have been reported also in other species [35-39]. Those reports, in accord with our results, demonstrate that the highest VEGF mRNA expression and VEGF protein concentration are detected during the early luteal phase, and are followed by a significant decrease of expression during the mid and late luteal phases.

Taken together, our results provide that in addition to intense angiogenesis (characterized by maximal VEGF concentration), elevated luteal gelatinases may contribute to the extensive luteal ECM and tissue remodelling that occurs as the postovulatory follicle is transformed into the CL [15,21]. Many studies relative to neoplastic growth well describe the interplay between VEGF and MMPs [40-43]. Bergers et al. [44] demonstrated that MMP9 is able to mobilize matrix attached VEGF isoforms and this action results essential for the switch between vascular quiescence to angiogenesis during carcinogenesis. Another study showed that VEGF regulated ovarian cancer invasion through secretion and activation of MMPs [45].

After the CL is fully formed, steroidogenesis is maximal during the midluteal period, and MMP activity and VEGF concentration are at basal levels. Interestingly, we previously reported an increased expression of the less abundant and matrix-attached VEGF splice variants (VEGF188 and VEGF182) as well as of the two VEGF receptors in this moment [5]. In this phase, luteal tissue prepares itself to the next step cumulating high levels of FPr and endonuclease making the tissue very sensitive to luteolytic stimulus. With the onset of structural regression, the MMPs are again called into action for the remodelling and removal of the CL [7].

The elevated expression of VEGF associated to the presence of MMP-9 at pregnancy suggests that initial angiogenic process during the early luteal phase may be renewed in swine pregnant CL. Increased angiogenesis at pregnancy in luteal tissue is controversial. Wulff et al. [46] demonstrated that luteal rescue is associated with a second wave of angiogenesis in human CL, while Rowe et al. [47] stated that no pregnancy-induced angiogenesis take place in marmoset CLs. Certainly, a stable and efficient vascular bed are required for the endocrine function of pregnant CL. Pauli et al. [48] reported that the administration of anti-VEGFR-2 antibody during the pre- and post-implantation periods in rodents, disrupted maternal ovarian function eliminating pre-existing luteal blood vessels. Thus, VEGF/VEGFR-2 pathway is critical to maintain the functionality of luteal blood vessels during pregnancy and might also be involved in regulating vascular permeability and P4 release into the bloodstream [49]. Therefore, the increase in VEGF at pregnancy is not unexpected but the increase in MMP-9 needs further clarifications. The different profile of VEGF/MMP-9 expression observed between fully formed (midluteal phase) and pregnant CLs should be taken into account when physiological effects will be monitored.

## Conclusion

We have demonstrated that CL development and regression is a very useful model for studying VEGF/MMPs relationships. During the very early luteal phase, high MMPs activities coupled with high VEGF levels drive the tissue to an "angiogenic phenotype", allowing CL growth under LH stimulus. In the late luteal phase, low VEGF and elevated MMPs levels may play role in the apoptotic tissue and ECM remodelling during structural luteolysis.

## Methods

### Animal model

Synchronized folliculogenesis was obtained in 28 prepubertal Large White gilts (96 ± 2.13 Kg, mean ± SEM) by administration of 1250 IU equine chorionic gonadotropin (eCG; Folligon, Intervet, Holland) and 750 IU hCG (Corulon, Intervet) 60 h later. In this model, ovulation occurs around 42–44 h after hCG administration (day 0). Four animals were artificially inseminated 40 h after hCG administration and pregnancies were determined by ultrasonography 35 days after. Ovaries were recovered by surgical laparotomy on days 1, 3, 5, 10, 14 and 17 after ovulation (n = 4 animals/time point) and at day 60 of pregnancy. Animals were pre-anesthetized by using azaperone (240 mg/gilt; Stresnil, Janssen, Belgium) and atropine sodium salt (2 mg/gilt; Industria Galenica Senese, Italy), and maintained under thiopental sodium (1.5 g/gilt; Pentothal Sodium; Gellini, Latina, Italy) anaesthesia. Five CLs from each gilt, chosen totally random from both ovaries, were isolated, cut in three parts with a razor blade and employed to perform all the analysis.

One third was weighed and homogenized in PBS (0.1 g/ml) on ice bath by an Ultra Turrax. The homogenate obtained was processed as follows: 500 μl were centrifuged at 2000 × g for 10 min at 4°C and supernatant was stored at -20°C until VEGF determination and the remainder of the homogenate was kept frozen until progesterone (P4) measurement and MMPs activity evaluation.

Total RNA was isolated from the second third of the CLs with the Tri-Pure reagent (Roche Diagnostic GmBH, Mannheim, Germany) and stored at -80°C until VEGF and Prostaglandin F2α receptor (FPr) mRNA levels quantification. The last part of samples was immediately processed for nuclei extraction and Ca^++^/Mg^++^-dependent endonuclease activity evaluation. All the sampling procedures were executed within 2 hours from the surgical removal of the ovaries.

All animals were housed and used according to EEC animal care guidelines. The experimental procedures had been previously approved by the Ethical Committee of Bologna University.

### P4 assay

Aliquots of 20 μl from each homogenate CL were extracted with 5 ml petroleum ether. After centrifugation, ether was collected and dried under a N_2 _stream. Dried ether extracts were resuspended in 1 ml phosphate buffer, diluted 1:50 and aliquots of 50 μl were then assayed by a validated RIA as previously described [9].

The sensitivity of the assay was 3.7 pg/tube. The intra- and interassay coefficients of variation were 6.3 and 19.6%, respectively. The results are expressed in ng/mg tissue.

### RNA extraction and Real-time quantitative RT-PCR

Total RNA from CLs, homogenized in Tri-Pure reagent (50 mg/ml), was extracted according to manufacturer's instructions (Roche Diagnostic GmBH, Mannheim, Germany). Purified RNA was resuspended in RNase-free water and quantified (A_260 _nm). One microgram of total RNA was reverse-transcribed to cDNA using iScript cDNA Synthesis Kit (Bio-RAD Laboratories Inc., CA, USA) in a final volume of 20 μl, according to the manufacturer's instruction. Transcription reactions without reverse transcriptase were performed to control for an eventual DNA contamination.

Swine primers were designed for VEGF, FPr and HPRT (Hypoxanthine Guanine Phosphorybosyl Transferase), using the Beacon Designer 3.0 Software (Premier Biosoft International, Palo Alto, Ca, USA). Their sequences, expected PCR product length and accession number are shown in Table 1. Real-time quantitative PCR was performed in the iCycler Thermal Cycler (Bio-RAD Laboratories Inc., Hercules, CA, USA) using SYBR green I detection. The following reaction components was prepared to the indicated end-concentrations: 0.6 μM of each primer, 1X IQ SYBR Green BioRad Supermix (Bio-RAD Laboratories Inc.), 150 ng of cDNA and H_2_O nuclease free to a final volume of 25 μl. All samples were performed in duplicate for all genes. The two step real-time PCR protocol employed was: initial denaturation for 3 min at 95°C, 40 cycles at 95°C for 15 sec and 60°C for 30 sec, followed by a melting step with a slow heating from 55 to 95°C with a rate of 0.05°C/s. The specificity of the amplified PCR products was verified by analysis of the melting curve, which is product-specific. The relative mRNA level was determined as the PCR cycle number that crosses an arbitrarily placed signal threshold (Ct). The Ct value correlates inversely with the amount of target mRNA in the sample. The housekeeping gene HPRT was used to normalize the amount of RNA. The relative changes in VEGF and FPr expressions were examined using the ΔΔCt method described previously [50], with ΔCt = Ct_target _- Ct_HPRT _and ΔΔCt = ΔCt_(days 3,5,10,14,17,60) _- ΔCt_(day 1)_. As PCR amplification is an exponential process, a ΔΔCt difference denotes a shift in regulation by a factor of two (2^-ΔΔCt^).

**Table 1:** Sense and antisense primers sequences used for real time RT-PCR.

Primer	Sequence (5'-3')	Product size (bp)
HPRT sense	GGACAGGACTGAACGGCTTG	
HPRT antisense	GTAATCCAGCAGGTCAGCAAAG	115
VEGF sense*	CCTTGCCTTGCTGCTCTACC	
VEGF antisense*	CGTCCATGAACTTCACCACTTC	101
FPr sense	TCAGCAGCACAGACAAGG	
FPr antisense	TTCACAGGCATCCAGATAATC	151

*VEGF primers were located on a common region for all VEGF isoforms.

Real-time efficiencies were acquired by amplification of a standardised dilution series and corresponding slopes and PCR efficiencies were calculated using iCycler iQ Real Time PCR Detection System (Bio-Rad Laboratories Inc.).

### Ca^++^/Mg^++^-dependent endonuclease activity assay

Tissues were immediately treated to isolate nuclei and to determine endonuclease activity in the samples [51].

In brief, minced tissue was homogenized (1/10, w/v) with a Dounce homogenizer in a homogenization solution containing 10 mM Tris-Cl (pH 7.4), 3 mM MgCl_2_, 3 mM EGTA, and 250 mM sucrose. The homogenate was filtered and then centrifuged at 800 g for 15 min at 4°C. The resulting pellet was resuspended in the homogenization solution supplemented with 0.5% (v/v) nonidet P40, incubated for 15 min at 4°C and then centrifuged at 800 g for 15 min at 4°C. The resulting pellet was resuspended in a solution containing 10 mM Tris-Cl (pH 7.4), 25 mM NaCl, and 340 mM sucrose. The reaction mixture for the DNA fragmentation assay was performed with 30 mg of luteal tissue and 1 mM Ca^++ ^and 5 mM Mg^++^, with or without the addition of 2 mM Zn^++^. A control reaction, without salts, was also carried out. The reactions were performed at 37°C for 10 min, after which low and high molecular weight DNA were extracted; residual RNA was removed by addition of RNase A. DNA content was evaluated by densitometric scanning under a UV transilluminator after 2% agarose gel electrophoresis run. Low molecular weight DNA (≤ 2000 bp) was expressed as percent of total DNA in the sample.

### VEGF assay

Luteal VEGF concentrations were measured in 100 μl samples of homogenate supernatants by a specific enzyme linked immune-adsorbent assay (ELISA, Quantikine, R&D Systems, Minneapolis, MN, USA) previously validated for the measurement of porcine VEGF [4]. This highly specific sandwich assay recognizes VEGF164 as well as VEGF120, while it exhibits negligible cross-reactivity with all cytokines/growth factors tested. A 96-well plate reader (Biomek 1000, Beckman Instruments, Fullerton, CA, USA) set to read at 450 nm emission was used to quantify the results. The sensitivity of the assay was 5 pg/ml, and the intra- and interassay coefficients of variation were less than 6 and 10%, respectively. All data are expressed as pg/mg protein; protein concentration was determined according to Lowry method [52], using a protein assay kit (Sigma Diagnostics, St Louis, MO, USA).

### MMPs activity assay

MMP-2 and -9 activities were analyzed by use of gelatin zymography on 10% Tris-Glycine poliacrylamide pre-cast gels with 0.1% gelatin (10% Novex Zymogram Gels, Invitrogen U.K.). Aliquots containing 30 μg of total proteins, mixed with an equal volume of sample buffer (Novex Tris-Glycine SDS sample Buffer 2X, Invitrogen U.K.) were loaded into the gel. Electrophoresis was performed under non-reducing conditions at a constant voltage (125 V for 120 minutes). Following electrophoresis, gels were washed for 30 minutes in Novex Zymogram Buffer (Invitrogen U.K.), equilibrated at room temperature for 30 minutes in developing buffer (Novexα Zymogram Developing Buffer, Invitrogen U.K.) and then incubated at 37°C for 22–24 hours in fresh developing buffer. Band of gelatinolytic activity were developed after staining gels for 6–8 hours with Simply Blue Safe stain (Invitrogen U.K.) by comparison with a MMP-2 and -9 human standard (Chemicon International, CA, USA).

Gel images were captured with a computerized system (Geldoc 1000, Bio-Rad), and gelatinolytic bands were measured with densitometric analysis software (Quantity One, Bio-Rad). The resulting data are expressed as arbitrary units (AU).

### Statistical analysis

The statistical analysis were based upon the mean of 5 CLs per animal (n = 4/time point), since no significant differences among CLs within the same animal were observed. Differences in relative mRNA expression of VEGF and FPr (using the ΔCt values), VEGF protein levels, progesterone contents and MMPs and Ca++/Mg++-dependent endonuclease activities were determined using one-way ANOVA (SPSS Version 13.0, Inc, Chicago, IL, USA), followed by the Duncan's post-hoc test. Data are presented as mean ± SEM. A value of p < 0.05 was considered significant.

## Authors' contributions

All authors participated in experimental design and collected biological material. LAR carried out RNA extraction and real-time RT-PCR. MET performed MMPs activity and VEGF assays. AZ carried out endonuclease activity and P4 assays. MLB was responsible for animal care and surgical procedures. MF conceived and supervised the study. LAR and MF wrote the manuscript. All authors read and approved the final manuscript.
